# Newly isolated bacteriophages show efficacy and phage-antibiotic synergy in vitro against the equine genital pathogens *Klebsiella pneumoniae* and *Pseudomonas aeruginosa*

**DOI:** 10.1186/s12917-025-04989-1

**Published:** 2025-10-03

**Authors:** Martin Köhne, Ronja Hüsch, Elisa Peh, Juliane Hirnet, Anna Tönissen, Mathias Müsken, Madeleine Plötz, Sophie Kittler, Harald Sieme

**Affiliations:** 1 Unit for Reproductive Medicine, Clinic for Horses, University of Veterinary Medicine, Foundation, Bünteweg 15, 30559 Hannover, Germany; 2 Institute of Food Quality and Food Safety, University of Veterinary Medicine, Bischofsholer Damm 115, 30173 Foundation, Hannover, Germany; 3 Central Facility for Microscopy, Helmholtz Centre for Infection Research – HZI, Inhoffenstraße 7, 38124 Braunschweig, Germany

**Keywords:** Bacteriophage, Genital infection, Horse, Biofilm, Non-antibiotic treatment

## Abstract

**Background:**

Bacterial infections of the genital tract are a severe problem in equine reproduction. Biofilms produced by *Klebsiella* (*K.*) *pneumoniae* and *Pseudomonas* (*P.*) *aeruginosa* present further concerns in such cases as they can limit the success of antibiotic treatments. Alternative treatment approaches are urgently needed for treating bacterial equine genital tract infections and thus, reduce antibiotic use. The present study reports on the bactericidal efficacy of both, novel *K. pneumoniae*- and *P. aeruginosa*-specific phages, in a biofilm model and in conjunction with antibiotic drugs as tested in vitro.

**Results:**

In total, 15 phages with lytic activity (*K. pneumoniae*: *n* = 6; *P. aeruginosa*: *n* = 9) were isolated and host ranges were determined. Four phages with a broad host range (*K. pneumoniae*: *n* = 3; *P. aeruginosa*: *n* = 1) were selected for further characterization, including electron microscopy and whole genome sequencing. Significant bacterial growth reduction was observed in planktonic killing assays at three multiplicities of infection (MOI 1, MOI 10, MOI 100), when the phages vB_KpnS_LmqsRe28-1, vB_KpnS_LmqsRe28-2, vB_KpnM_LmqsRe27-1 or vB_PaeS-LmqsRe25-1 were added. In addition, planktonic killing assays were used to examine the four phages in conjunction with gentamicin, enrofloxacin and trimethoprim-sulfadiazine in vitro. The results indicate synergistic activities between the four phages and the investigated antibiotics. However, bacterial growth was not affected in biofilm models after phage treatment, likely due the absence of depolymerase genes in the sequenced phages.

**Conclusions:**

Phages against the equine genital pathogens *K. pneumoniae* and *P. aeruginosa* were evaluated in vitro for potential clinical use. While the bactericidal activity of the isolated phages was demonstrated in liquid culture, none of the tested phages showed significant reduction in in vitro biofilm models. However, due to synergistic activities between phages and antibiotics, further studies on combinatorial approaches are needed to develop strategies for managing difficult infections.

**Supplementary Information:**

The online version contains supplementary material available at 10.1186/s12917-025-04989-1.

## Background

Equine genital tract infections are a major problem in equine reproduction [1]. Chronic infectious endometritis is observed in up to 25–60% of barren mares and results in reduced pregnancy rates and economic losses [2–4]. Among bacterial pathogens capable of infecting the genital tract, the gram-negative, facultative pathogens *Klebsiella* (*K.*) *pneumoniae* and *Pseudomonas* (*P.*) *aeruginosa* are the most concerning in horse reproduction and cannot easily be eradicated due their ability to form biofilms and their resistance to antibiotic treatment [5]. These bacteria frequently cause genital infections in mares [6, 7], in stallions [8, 9], and are often found in hospitalized horses [10]. Recently, the importance of rather unspecific environmental infections by venereal strains of both examined bacterial species has been elucidated in a review, demonstrating the risk of infections with unspecific but virulent environmental strains [11]. Antimicrobial resistance (AMR) has increased worldwide – especially in gram-negative bacterial species such as *P. aeruginosa* and *K. pneumoniae* – posing a threat to human and animal health [12]. Although the number of antibiotic drug resistant genital tract infections in horses is currently reported to be low, first reports on infections with ESKAPE pathogens (*Enterococcus faecium*, *Staphylococcous aureus*, *K. pneumoniae*, *Acinetobacter baumannii*, *P. aeruginosa*, and *Enterobacter* spp.) in horses are concerning for equine genital health [13–16].

The persistence of *K. pneumoniae* and *P. aeruginosa* can also be attributed to their ability to form biofilms [17], protecting the bacterial cells against the host immune system [18], as well as antibiotic treatment [19]. Biofilms usually adhere to surfaces and consist of a complex extracellular matrix, composed of nucleic acids, proteins, polysaccharides and lipids as well as bacteria with reduced metabolic and replication rates relative to free planktonic bacteria [20, 21]. Consistent with the finding that biofilms might occur in uterine infections, traditional antibiotic treatments in mares show reduced efficiency, leading to reoccurring infections [22].

The biofilm-forming abilities of *Pseudomonas* and *Klebsiella* in equine genital infections and concerning AMR rates highlight the need for alternatives to antibiotics for treatment. One interesting approach is the use of bacteriophages. Bacteriophages (phages) are ubiquitous viruses that infect and kill bacterial cells [23] and have been used in antimicrobial treatments for more than 100 years [24]. Phages are bactericidal and remain concentrated at the site of an infection, since their activity and replication depend on the presence of specific host bacteria. In recent years, their therapeutic potential has drawn attention due to increasing rates of infection by multi-drug resistant (MDR) bacteria [25]. They pose promising future therapeutics based on their remarkable potential that has been reported in various studies [26, 27]. Moreover, some phages express enzymes with the capacity to weaken the extracellular matrix of biofilms, improving success of antimicrobial drugs applied to combat the infection [28]. However, the phages’ immunogenicity and their selectivity to certain bacterial strains poses some challenges for the successful clinical application of phage therapy [29]. To date, there are no case reports and clinical studies on the therapeutic use of phages in equine medicine. Experimental rodent models of equine diseases have been published by Furusawa et al., who successfully administered *P. aeruginosa-*specific phages in a mouse model of equine keratitis [30]. Another study investigated the use of phages specific to *Salmonella* Abortusequi in a mouse model [31].

Based on the lack of phage studies in equine medicine, the aims of the present study were (i) to isolate and characterize specific phages against the important equine genital pathogens *P. aeruginosa* and *K. pneumoniae* and (ii) to determine their bactericidal capacities alone and (iii) in combination with selected antibiotic drugs in vitro as well as (iv) in a biofilm model to prepare for clinical studies.

## Materials and methods

### Bacterial isolates

*K. pneumoniae* (*n* = 26) and *P. aeruginosa* (*n* = 33) isolates obtained in samples collected in horses (Supplementary material: Table 1) were provided by the Institute of Microbiology, University of Veterinary Medicine Hannover, Foundation and Labor Dr. Böse, GmbH, Harsum, Germany. After storage in cryotubes (Carl Roth GmbH + Co. KG, Karlsruhe, Germany) at −80 °C before the onset of experiments, bacterial isolates were streaked onto Columbia agar supplemented with 5% sheep blood (Oxoid Deutschland GmbH, Wesel, Germany) and incubated at 37 °C overnight.

The study was approved as ethical by an institutional review board (Doctoral Commission, Stiftung Tierärztliche Hochschule Hannover, 2023, 3.5) and the animal welfare officer (TVO-2023-V-18).

### Isolation and propagation of phages

The soft-agar overlay technique was used to detect *Pseudomonas-* and *Klebsiella*-specific phages as described previously [32, 33]. Phages were isolated from samples of different origins: horses (uterine lavage fluid; *n* = 1), horses’ environment (manure, uterine lavage fluid, drain water of horse husbandries; *n* = 11) and sewage water (*n* = 2). Samples (approximately 2 g) were dispersed in falcon tubes (50 mL) in sodium chloride-magnesium sulfate (SM) buffer (100 mM NaCl, 8 mM MgSO4, 50 mM Tris-HCl (pH 7.5)). After shaking overnight at 4 °C and 120 rpm, the dispersed samples were centrifuged (5000 × *g*, 20 min, 4 °C) and the supernatant was filtered through a 0.2 μm polyethylene sulfone membrane (PES) syringe filter (Carl Roth GmbH + Co. KG). For this purpose, overnight cultures of *P. aeruginosa* (*n* = 21) and *K. pneumoniae* isolates (*n* = 4) were prepared by plating out the bacteria onto Columbia agar supplemented with 5% sheep blood (Oxoid Deutschland GmbH) and incubating at 37 °C for 18 h. Subsequently, grown colonies were suspended in 10 mM MgSO_4_ and adjusted to a turbidity in accordance with McFarland standard 3.0. Equal volumes (100 µL) of the bacterial suspension and of filtered dispersed samples were mixed in 5 mL 0.7% LB soft agar (Carl Roth GmbH + Co. KG). Next, the mixture was given onto LB base agar (1.5%) plates. After solidifying, double agar-overlays were incubated for 24 h at 37 °C. After incubation, the bacterial lawn was examined for plaques – small clearings in the bacterial lawn in which the cells were lysed by phages. For isolation and purification of phages, a successive 3-fold (*Klebsiella* phages) or 8-fold (*Pseudomonas* phages) picking and plating procedure of single plaques was performed. Before storage (4 °C) until further use, phages were propagated to obtain concentrations of 10^8^ – 10^9^ PFU/mL. Phage titers were determined by plating 100 µL of a 10-fold serial dilution series of the phage suspension on the respective host bacterial isolate using the soft agar-overlay technique.

### Determination of host range and efficiency of plating

The susceptibility of 26 *K. pneumoniae* isolates and 33 *P. aeruginosa* isolates towards the isolated phages (*K. pneumoniae*-specific: *n* = 6 and *P. aeruginosa*-specific: *n* = 9) was determined using a conventional spot test combined with an efficiency of plating (EOP) assay as previously described [33]. In brief, 1 mL of bacterial suspension (*P. aeruginosa*: *n* = 33; *K. pneumoniae*: *n* = 26) was transferred to 9 mL LB broth and incubated for two hours on a shaking platform (120 rpm) at 37 °C. Subsequently, 100 µL of the bacterial cultures were mixed into molten 0.7% agar and poured onto LB base agar plates. After solidifying, 10 µL of 10-fold dilution series were spotted on the overlay for each phage. After 18 h at 37 °C, the overlay plates were evaluated. Plaque counts were used to calculate the EOP by dividing the concentration on the target isolate by the concentration measured on the host strain of the respective phage. All experiments were repeated three times.

The relative efficiency of plating was ranked according to the presence of an inhibitory zone or number of plaques detectable on the agar plates: no sensitivity (no plaque formation), very low sensitivity (presence of a zone of inhibition), moderately low sensitivity (presence of one opaque plaque), low sensitivity (EOP < 0.1), moderate sensitivity (0.1 ≤ EOP ≤ 1), high sensitivity (1 < EOP ≤ 10) and ultimate sensitivity (10 < EOP). Ultimate sensitivity was similar to the sensitivity observed in the original host bacterial isolate.

### Phage DNA extraction, whole-genome sequencing, and bioinformatic analysis

Phages (*n* = 4) were selected based on their host range and phage suspensions (300 mL, 10^8^−10^10^ PFU/mL) were prepared as described above for further characterization. Next, suspensions were centrifuged in a high-speed centrifuge for concentrating the phage particles (24,000 × *g*, 2 h, Avanti J-26 S XP, Beckmann Coulter Inc., Brea, CA, USA). The resulting pellet was resuspended in SM-buffer and a purification step by cesium chloride-gradient in Tris buffer (gradient: 1.3 to 1.6 g/mL CsCL; 165,100 × *g*, 4 °C, 2 h, Optima XPN-100, Beckmann Coulter Inc.) was performed. The dialysis was performed to exchange buffer from CsCl (containing Tris) to SM buffer. The resulting phage suspensions were used for electron micrographs and DNA isolation. For extraction of phage DNA from the purified virions treated with Norgen’s RNase-Free DNase I Kit (Norgen Biotek Corp., Thoroid, Canada), a Phage DNA Isolation Kit (Norgen Biotek Corp.) was used according to the manufacturer’s instructions. Phage DNA concentration was determined with the dsDNA HS Assay Kit (Life Technologies Corporation, Oregon, USA) before submitting the isolated DNA to whole genome sequencing with a Nextseq sequencing system (Microsynth AG, Balgach, Switzerland). Additional MinION sequencing of vB_KpnS_LmqsRe28-1 and vB_KpnS_LmqsRe28-2 was performed on a Minion in a Min114 flowcell, library preparation with ligation the sequencing kit V14. The average read lengths were: vB_PaeS_LmqsRe25-1 100 bp (Illumina), vB_KpnM_LmqsRe27-1 100 bp (Illumina), vB_KpnS_LmqsRe28-1 400 bp (Minion) and vB_KpnS_LmqsRe28-2 100 bp (Illumina)/920 bp (Minion). The Galaxy platform (version 24.2; [34]) was used for assembly and annotation. Assembly of contigs was performed with the SPAdes software (St. Petersburg genome assembler; version: 3.15.5) and BLASTN (Basic Local Alignment Search Tool, http://www.ncbi.nlm.nih.gov Version 2.15.0) was run to detect homology with previously published phages. Long reads were cleaned using filtlong (https://github.com/rrwick/Filtlong Version 0.2.1), assembled using Flye, and short reads were mapped on the assembly using BWA-MEM [35] and polished with pilon. Alignment and phylogenetic tree were generated based on the genome sequence of phages using ClustalOmega. Prokka (Prokaryotic genome annotation; Galaxy Version 1.14.6) and Pharokka (rapid standardized annotation tool for phage genomes and metagenomes; Galaxy Version 1.3.2) were used for annotation of CDS. The tool Phastest [36] was used to independently confirm the annotation obtained with Pharokka. Searching for depolymerases, Phage DPO was used on the Galaxy Docker build (https://galaxy.bio.di.uminho.pt/). Phage termini were determined using PhageTerm [37] and confirmed by PCR as described by Boeckman et al. [38].

### Electron microscopy

According to Dreiseikelmann et al. [39], phages were absorbed for 15–30 s onto a carbon film and washed twice on TE buffer droplets (10 mM TRIS, 1 mM EDTA, pH 6.9). After the washing steps, negative staining of the samples was conducted with 2% aqueous uranyl acetate by heat dying on a bulb (60 W) after removing excessive liquid with a filter paper. A Zeiss TEM 910 transmission electron microscope (Zeiss, Oberkochen, Germany) at an acceleration voltage of 80 kV and at calibrated magnifications with a line replica was used for examination of samples. A slow-scan closed circuit device (CCD)-camera (ProScan, 1,024 × 1,024, Proscan Elektronische Systeme GmbH, Scheuring, Germany) with ITEM-Software (version 5.0 (build 1210); Olympus Soft Imaging Solutions GmbH, Münster, Germany) recorded the images digitally. The same software was used to measure the head diameter and tail length from five measurements. The average sizes were calculated with Excel.

### One step growth experiments

One step growth experiments were performed as previously described [32] with some modifications. In brief, selected phages were incubated at 37 °C with their respective host isolates. The cultures of the host bacteria were grown to an OD_600_ of 0.25 and mixed with either vB_PaeS_LmqsRe25-1, vB_KpnS_LmqsRe28-1, vB_KpnM_LmqsRe27-1 or vB_KpnS_LmqsRe28-2 at a multiplicity of infection of 0.02. After a co-incubation of phages and their target bacteria for 10 min at room temperature, samples were centrifuged at 1,300 × *g* for 4 min and the determined phage concentration in the supernatant, containing the free phages, was used to calculate the number of phages adsorbed to bacteria. The pellets were resuspended in fresh LB and placed in a heating block (37 °C). Samples were withdrawn, and concentrations of phages in the samples were determined immediately at 10-min intervals for up to 2 h incubation. Experiments were performed in duplicate and were repeated thrice. The latent period and burst size were calculated as described [32].

### Planktonic killing assay of phages and antibiotics for synergy testing

The antibiotic susceptibility profiles were determined using minimum inhibitory concentration (MIC) methods in accordance with the specifications given in the Clinical and Laboratory Standards Institute (CLSI) document VET01, CLSI Vet01S 6th ed., CLSI Vet06, CLSI Vet09 and CLSI M100S. Susceptibility testing was performed by the Institute of Microbiology, University of Veterinary Medicine, Foundation, Hannover, Germany. The MIC value was defined as the lowest concentration of an antibiotic agent that inhibited visible growth of bacteria. Based on the results, the antibiotic concentrations (MIC, 0.5 MIC, 0.25 MIC) were chosen for the subsequent experiments.

Second, planktonic killing assays were performed in which phages and antibiotics were used individually and in combination to evaluate possible synergistic interactions. Bacterial isolates and their respective phages (vB_KpnS_LmqsRe28-1 (Isolate 2), vB_KpnS_LmqsRe28-2 (Isolate 3), vB_KpnM_LmqsRe27-1 (Isolate 12), PaeS_LmqsRe25-1 (Isolate 29)) were selected for the experiments according to their host ranges. For synergy testing, gentamicin (Gentamycinsulfat, Serva Electrophoresis GmbH, Heidelberg, Germany), enrofloxacin (Enrofloxacin, ≥ 98%, MP Biomedicals™, Eschwege, Germany) and trimethoprim-sulfadiazine (Trimethoprim-Lactate, 98%; Sulfadiazine, 99%, ThermoScientific Chemicals, Schwerte, Germany) were used in three different concentrations (MIC, 0.5 MIC, 0.25 MIC as described above). The planktonic killing assays were conducted similar to methods described by Peh et al. [40] with some modifications.

In brief, the phage solutions were prepared at three different concentrations (2 × 10^6^ PFU/mL; 2 × 10^7^ PFU/mL; 2 × 10^8^ PFU/mL) in Standard Nutrient Broth I (Carl Roth GmbH + Co. KG) to obtain different multiplicities of infection in the wells (MOI 1, MOI 10, MOI 100). Two-fold dilution series of gentamicin, enrofloxacin and trimethoprim-sulfadiazine were prepared accordingly to achieve final concentrations of MIC, 0.5 MIC and 0.25 MIC in the wells. Bacterial overnight cultures were adjusted to a McFarland standard of 0.5 in 10 mM MgSO_4_ and diluted 1:100 in Standard Nutrient Broth I (broth).

U-shaped bottom 96-well plates (Sarstedt AG Co. KG, Nürnbrecht, Germany) were used. Wells were prepared as follows: (i) for positive controls, wells were filled with 100 µL bacterial suspension and 100 µL broth; (ii) for testing phages or antibiotics alone, 50 µL of the respective agent (phage or antibiotic), 50 µL broth and 100 µL bacterial suspension were added to the wells; (iii) for testing phages and antibiotics in combination, 50 µL of the respective phage dilution, 50 µL of the antibiotic and 100 µL bacterial suspension were added to the wells; (iv) for negative control, 200 µL broth were added to the wells. An exemplary plate assignment can be found as supplementary material (Supplementary Material: Table 2).

The microtiter plate was incubated in a Tecan Spark automatic plate reader (Tecan Group AG, Männedorf, Switzerland) with double orbital shaking at 37 °C for 24 h. Optical density (OD_600_) was measured hourly during incubation. All experiments were performed at least in triplicate.

The area under the curve (AUC) of OD_600_ measurements was calculated according to Peh et al. [40]:

$$\:AUC=\sum\:_{h=1}^{24}\frac{ODh+1+ODh}{2}$$

To compare bacterial growth in the presence and absence of phages and/or antibiotics, the virulence indices (vi) were calculated as follows: $\:vi\:\left[\%\right]=100-(\left(\frac{AUCphage}{AUCpositivecontrol}\right)\times\:100)$

### Efficacy testing of phages to reduce K. pneumoniae and P. aeruginosa biofilms in vitro

Efficacy of the phages vB_KpnS_LmqsRe28-1 (LmqsRe28-1), vB_KpnS_LmqsRe28-2 (LmqsRe28-2) and vB_KpnM_LmqsRe27-1 (LmqsRe27-1, specific to *K. pneumoniae*) as well as vB_PaeS_LmqsRe25-1 (LmqsRe25-1, specific to *P. aeruginosa*) was determined in a biofilm model as described by Elgamoudi and Korolik [41] with some modifications. Briefly, bacterial suspensions (*K. pneumoniae* isolates 2, 3 and 12; *P. aeruginosa* isolate 29) were prepared in 0.9% NaCl solution and adjusted to a McFarland standard of 0.5 (1 × 10^8^ CFU/ml). For biofilm production, 5 µL of the bacterial suspensions were added to 195 µL LB and filled into wells of a U-shaped bottom 48-well plate (Sarstedt AG Co. KG) and incubated for 48 h at 37 °C and 120 rpm in a humid environment, achieved by placing a small container of water and a stack of wet paper towels around the plate during incubation.

Biofilm formation (optical density and bacterial growth) was evaluated after 48 h by discarding the supernatant and washing the plate twice with PBS. For analysis of bacterial growth, PBS (200 µL) was added and the biofilm was removed with a sterile loop. After repeated pipetting (5-fold) to dissolve bacterial aggregates, dilution series were plated on LB-agar as duplicates. Concentrations were determined by using the following formula:

$$\:\frac{total\:no.\:\:of\:CFU\:\left(all\:plates\right)}{no.\:\:of\:plates\:\left(lowest\:dilution\right)*1+\:no.\:\:of\:plates\:\left(2nd\:lowest\:dilution\right)*\text{0,1}}=concentration\:[CFU/ml]$$

For optical density measurements, biofilms were stained using crystal violet (125 µL of 0.1% crystal violet per well) and incubated for 10 to 15 min. After two consecutive washing steps with PBS, plates were dried over night at room temperature. Subsequently, crystal violet was removed by adding 125 µL acetic acid (30%) per well and incubating the plates for 10 to 15 min. Afterward, the dissolved biofilms were transferred to a new 48-well-microplate and optical density (OD_600_) was measured with a Tecan Spark Plate Reader. Duplicate wells filled with 125 µL acetic acid (30%) served as negative controls.

For efficacy testing of phages in dispersing biofilm mass and reducing bacterial growth, established biofilms (48 h) were inoculated with the respective phage suspension (100 µL) in two different multiplicities of infection (MOI 1 and MOI 10) after a single washing step with PBS. Negative and positive controls were prepared by addition of 100 µL LB. All plates were incubated under humid atmosphere for 6 and 24 h, respectively. After 6 and 24 h, bacterial growth and optical density were determined as described above for different aliquots.

### Statistical analysis

Statistical analysis of the data was performed using GraphPad Prism 9.2.0 (GraphPad Software, San Diego, USA). Data on bacterial concentration was transformed to log_10_ CFU/mL. For parametric data (Shapiro-Wilk test), unpaired t-tests were conducted. Dunn’s multiple comparison test was used for analyzing data from efficacy testing of phages in liquid culture and in biofilms for significant differences. The level of significance was set at *p* < 0.05.

## Results

### Isolation and characterization of phages

Overall, the phages were detected in 14 samples from horses, horse husbandries and sewage water plants using the soft-agar overlay method with clinical isolates of *P. aeruginosa* (*n* = 21) and *K. pneumoniae* (*n* = 4). After purifying the phages by serial propagation of plaques (*K. pneumoniae*-phages 3 rounds of picking and plating; *P. aeruginosa* 8 rounds of picking and plating, due to the smaller size of the plaques), six *K. pneumoniae*- and nine *P. aeruginosa*-specific, purified phages were received as shown in Table 1.

**Table 1:** Overview on isolated phages with name, origin, bacterial isolate number, bacterial host and isolation passage. Head sizes and tail lengths were determined with electron microscopic imaging for selected phages

Phage name	Origin	Host bacterial isolate	Isolation passage	Head size (nm)	Tail length (nm)
vB_Kpn_LmqsRe28-3	wastewater after biological purification	*K. pneumoniae* 1	4	-	-
vB_Kpn_LmqsRe28-5	wastewater after biological purification	*K. pneumoniae* 12	4	-	-
vB_Kpn_LmqsRe28-4	wastewater after biological purification	*K. pneumoniae* 12	4	-	-
vB_KpnS_LmqsRe28-1	wastewater after biological purification	*K. pneumoniae* 2	4	58 ± 2.02	162 ± 0.71
vB_KpnS_LmqsRe28-2	wastewater after biological purification	*K. pneumoniae* 3	4	75 ± 4.19	184 ± 3.54
vB_KpnM_LmqsRe27-1	wastewater after mechanical purification	*K. pneumoniae* 12	4	57 ± 3.55	18 ± 1.55
vB_PaeS_LmqsRe25-1	horse manure	*P. aeruginosa* 29	8	62 ± 2.59	191 ± 3.30
vB_Pae_LmqsRe26-7	smegma (stallion)	*P. aeruginosa* 29	8	-	-
vB_Pae_LmqsRe234-1	uterine lavage fluid (pyometra)	*P. aeruginosa* 29	8	-	-
vB_Pae_LmqsRe235-2	drain (breeding barn)	*P. aeruginosa* 29	8	-	-
vB_Pae_LmqsRe236-1	drain (breeding barn)	*P. aeruginosa* 29	8	-	-
vB_Pae_LmqsRe237-2	drain (wash box)	*P. aeruginosa* 29	8	-	-
vB_Pae_LmqsRe238-2	drain, stallion stable	*P. aeruginosa* 29	8	-	-
vB_Pae_LmqsRe239-1	residues breeding barn	*P. aeruginosa* 29	8	-	-
vB_Pae_LmqsRe240-1	horse manure	*P. aeruginosa* 29	8	-	-

**Fig. 1:**
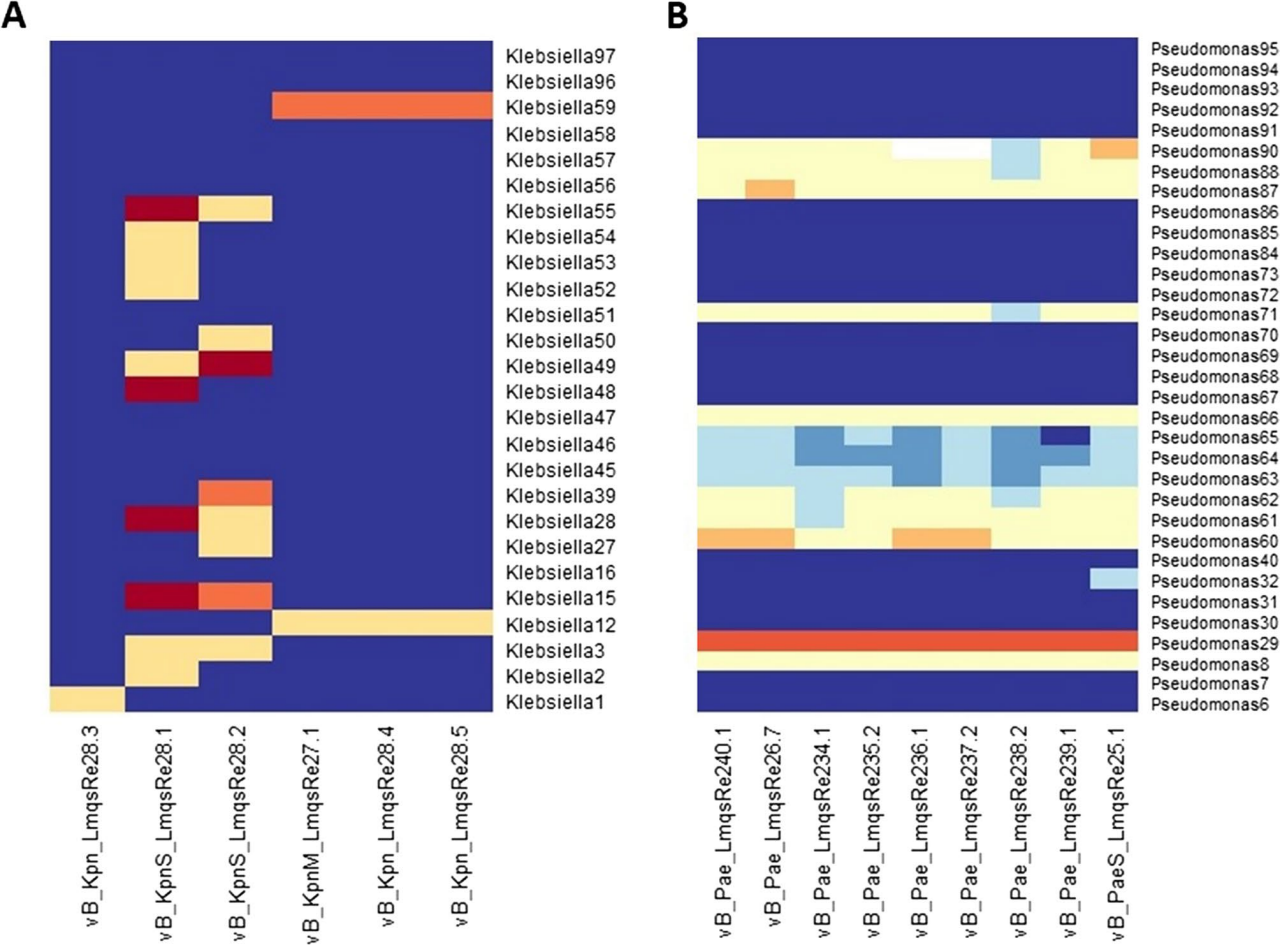
Heat maps of phage host ranges (**A**) *Klebsiella pneumoniae*; (**B**) *Pseudomonas aeruginosa*. Phages are displayed on the x-axis and bacterial isolates on the y-axis. Dark blue: no sensitivity (no plaque formation), medium blue: very low sensitivity (presence of a zone of inhibition), light blue: moderately low sensitivity (presence of 1 opaque plaque), low sensitivity (EOP < 0.1), pale yellow: moderate sensitivity (0.1 ≤ EOP ≤ 1), orange: high sensitivity (1 < EOP ≤ 10), red: ultimate sensitivity (10 < EOP); white: no evaluable result

The host ranges of all purified phages were determined using bacterial isolates of their respective host species (*P. aeruginosa*: *n* = 33; *K. pneumoniae*: *n* = 26) as shown in Fig. 1. The *P. aeruginosa* phages covered 44% of the 33 isolates tested. Phage LmqsRe25-1 had the broadest host range among the tested phages, forming plaques on 14 of 33 clinical isolates. In total, the *K. pneumoniae*-specific phages covered 16 (61%) of the 26 clinical isolates tested. For *K. pneumoniae*-specific phages, the broadest host range was displayed by LmqsRe28-1, forming plaques on 10 of 26 clinical isolates. The efficiency of plating (EOP) was highest for LmqsRe28-1, showing higher efficiency than in the host in four tested isolates (10 < EOP). In contrast, *P. aeruginosa*-specific phages displayed low EOP values on non-host isolates, achieving rather single plaque formation (EOP < 0.1 to EOP 1) than complete lysis (Fig. 1).

Four phages with a broad host range (in brackets) were used for further characterization (*P. aeruginosa*: LmqsRe25-1 (14/33), *K. pneumoniae*: LmqsRe28-1 (10/26), LmqsRe28-2 (8/26), LmqsRe27-1 (2/26)). All selected *Klebsiella* phages formed clear plaques with a halo on host strains, and plaque diameters ranged from 1 mm (LmqsRe28-1, LmqsRe28-2) to 4 mm (LmqsRe27-1). In contrast, the selected *Pseudomonas* phage formed clear plaques with a diameter less than 1 mm on host strains (Supplementary Material Fig. 1A-D). Detailed information on the phages’ burst sizes (ranging from 0.03 ± 0.01 PFU/cell (LmqsRe25-1) to 47.7 ± 41.99 PFU/cell (LmqsRe27-1)) and the respective one-step growth curves (latent period range from 20 min (LmqsRe27-1) to 40 min (LmqsRe25-1)) can be found in the supplementary material (Supplementary Material Fig. 2A-D).

**Fig. 2:**
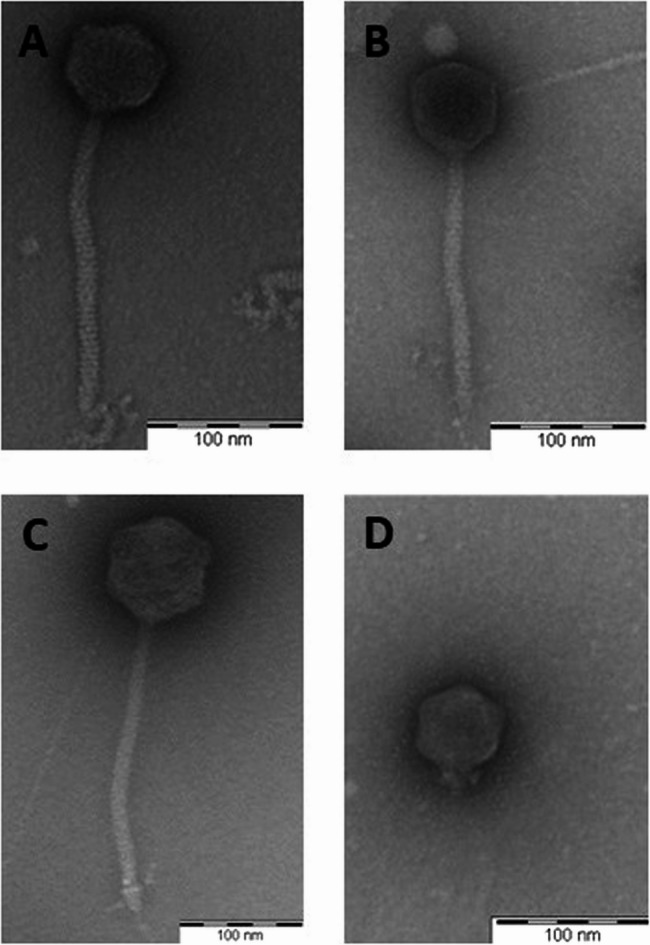
Transmission electron micrograph of negatively stained phages vB_PaeS_LmqsRe25-1 (**A**), specific to *Pseudomonas aeruginosa;*, vB_KpnM_LmqsRe28-1 (**B**), vB_KpnS_LmqsRe28-2 (**C**) and vB_KpnM_LmqsRe27-1 (**D**), B-D: specific to *Klebsiella pneumoniae*. Scale bar = 100 nm

Negatively stained electron micrographs of all four phages displayed phage particles with icosahedric heads and a tail (Fig. 2). The tails of LmqsRe25-1, LmqsRe28-1 and LmqsRe28-2 were long and non-contractile, as is typical for siphovirus morphology, while the tail of LmqsRe27-1 showed podovirus-like morphology.

**Fig. 3:**
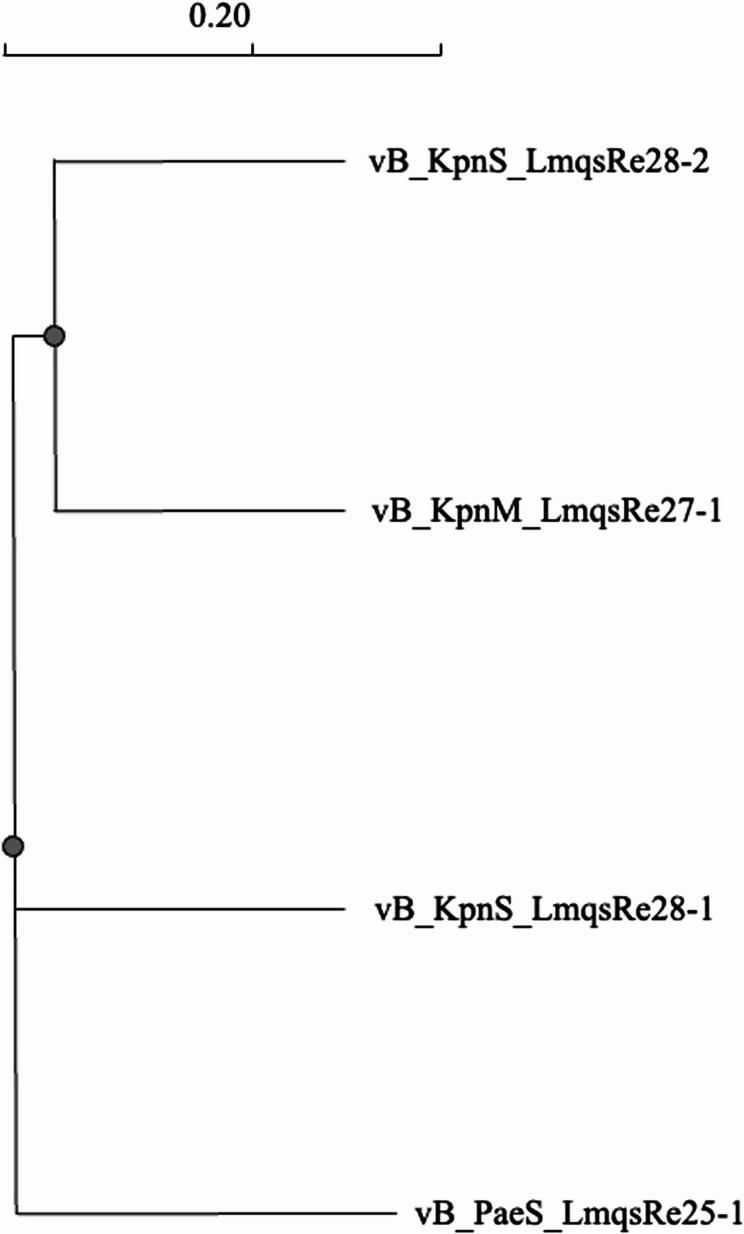
Phylogenetic analysis of newly isolated phages vB_KpnM_LmqsRe27-1, vB_KpnS_LmqsRe28-1, vB_KpnS_LmqsRe28-2 and vB_PaeS_LmqsRe25-1 (generated with ClustalOmega)

The selected phage genomes were sequenced on an Illumina platform and the resulting contigs were assembled using SPAdes for further analysis. Genomes showed lengths of 33,549 bp for LmqsRe25-1 and 40,706 bp for LmqsRe27-1. LmqsRe27-1 showed 94.3% identity with KP32i196 *Przondovirus* belonging to T7-like podoviruses [42]. No lysogeny-related genes or virulence genes were identified. Despite of several Illumina sequencing attempts for LmqsRe28-1, no reorganized genome was received using PhageTerm due to critically low coverage (29.6%). Thus, based on short reads only a partial genome could be assembled for LmqsRe28-1 and 28 − 2. However, another approach using longread nanopore technology to generate a scaffold for the short reads resulted in full genome sequences [43] that lacked lysogeny-related genes such as integrase or repressor genes. LmqsRe28-1 showed 99.1% identity to the non-classified *Caudoviricetes Klebsiella* phage P61_2 and an almost similar genome length of 48,177 bp. LmqsRe28-2 showed a genome length of 113,167 bp and 96.1% identity to the *Klebsiella* phage vB_Kpn2-P1 with similar genome size. *Pseudomonas*-specific phage LmqsRe25-1 showed 86% query coverage and 97.6% sequence identity to the recently published unclassified bacterial virus *Pseudomonas* phage Meadow [44]. Prokka analysis revealed the presence of the IS3 family transposase IS222 and lexA repressor genes and Pharokka the presence of a transcriptional regulator and excisionase gene. The bioinformatics analysis did not show the presence of genes that code for depolymerases in any of the phages. A dendrogram showing the relatedness of the phages is shown in Fig. 3. Surprisingly, the *Klebsiella* phage LmqsRe28-2 showed closer relatedness to LmqsRe27-1 than to the morphologically and phenotypically related phage LmqsRe28-1 in the phylogenetic analysis. The complete genome sequences of LmqsRe27-1, LmqsRe28-1 and LmqsRe28-2 were deposited into the GenBank database with the accession numbers PV660644 (LmqsRe27-1), PV660645 (LmqsRe28-1) and PV759140 (LmqsRe28-2). The genome sequence of LmqsRe25-1 was also deposited into the GenBank database (PX050560).

### Planktonic killing assays reveal synergistic interactions between phages and the tested antibiotics

**Fig. 4:**
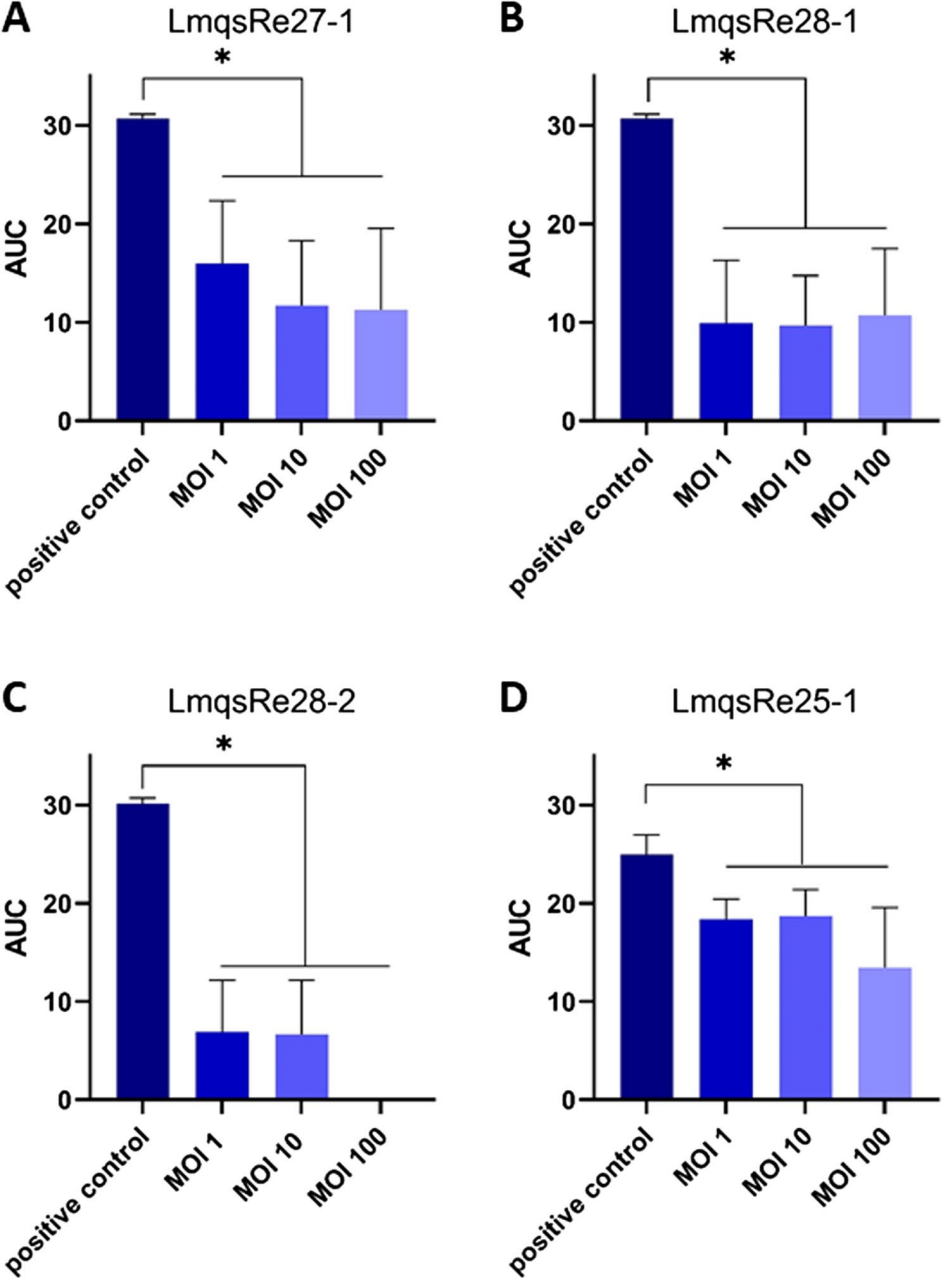
Area under the curve after incubating *Klebsiella pneumoniae* (*n* = 2; A: isolate 12, B-C: isolate 2) or *Pseudomonas aeruginosa* isolate 29 (*n* = 1; **D**) with different phages (**A**) vB_KpnM_LmqsRe27-1, **B**) vB_KpnS_LmqsRe28-1, **C**) vB_KpnS_LmqsRe28-2 and **D**) vB_PaeS_LmqsRe25-1 at different multiplicities of infection (MOI). * indicates a significant difference as determined by Kruskal-Wallis-test and Dunn’s posthoc test

A planktonic killing assay was performed to determine the inhibitory ability of phages and combinations of phages and antibiotics in liquid culture. Therefore, bacterial host strains were incubated alone, as controls, or together with phages at different multiplicities of infection (MOIs) and/or antibiotics. All tested phages were able to reduce bacterial growth of *K. pneumoniae* and *P. aeruginosa* significantly at multiple MOIs, (Fig. 4A-D) as determined by OD_600_ measurements. The phage virulence indices ranged from 100% (LmqsRe28-2; MOI 100) to 25% (LmqsRe25-1; MOI 10; Table 2). Bacterial growth was delayed in all cases for at least 8 h (LmqsRe27-1, LmqsRe28-1, and LmqsRe25-1; Supplementary Material Fig. 3A, B, D), and growth suppression lasted up to 12 h in the presence of LmqsRe28-2 (Supplementary Material Fig. 3C) not reaching final ODs as the respective controls.

**Table 2:** Virulence indices as determined by planktonic killing assays against *K. pneumoniae* and *P. aeruginosa* in different multiplicities of infection (MOIs)

Phage	Multiplicity of infection	Virulence index*[%]
vB_KpnS_LmqsRe28-1	1	68
	10	69
	100	65
vB_KpnS_LmqsRe28-2	1	77
	10	78
	100	100
vB_KpnM_LmqsRe27-1	1	48
	10	62
	100	63
vB_PaeS_LmqsRe25-1	1	26
	10	25
	100	46

**Fig. 5:**
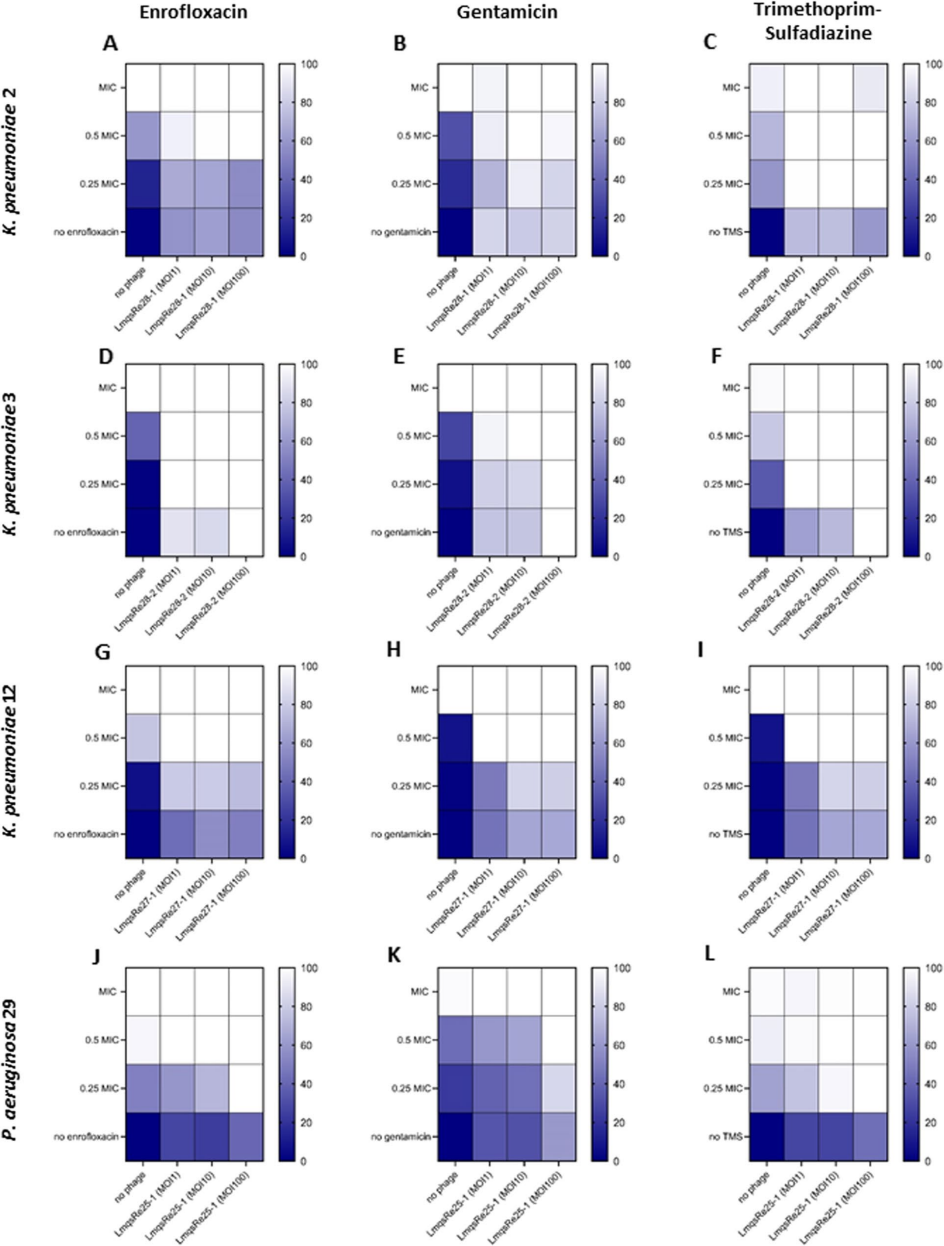
Synograms of phage-antibiotic interactions on growth of four different host bacteria (*K. pneumoniae* 2 (**A**-**C**), *K. pneumoniae* 3 (**D**-**F**), *K. pneumoniae* 12 (**G**-**I**) and *P. aeruginosa* 29 (**J**-**L**)). The area under the curve (AUC) of the bacterial isolates after incubation (24 h, 37 °C) with their respective phages at different multiplicities of infection (MOIs tested: no phage, MOI1, MOI10, MOI100) and antibiotic agents (enrofloxacin, gentamicin, trimethoprim-sulfadiazine (TMS)) with the full minimum inhibitory concentration (MIC) down to absence of antibiotic (no antibiotic) in decreasing concentrations (0.5 MIC, 0.25 MIC) was used for calculation of growth reduction. Complete absence (100% reduction) of bacterial growth is indicated by a white cell, no reduction (0%) reduction by a dark blue cell

To evaluate phage-antibiotic synergistic action on bacterial growth, we exposed host bacteria to a combination of different MICs (MIC, 0.5 MIC and 0.25 MIC) and MOIs over time in the above-mentioned setup. The synograms [45] of each phage with its respective host and the three different antibiotic agents are shown in Fig. 5A-L. The tested phage-antibiotic combinations varied widely in their efficacy. For LmqsRe28-1, synergistic effects were observed with gentamicin and trimethoprim-sulfadiazine and less pronounced also with enrofloxacin. When using phage LmqsRe28-2, enrofloxacin and trimethoprim-sulfadiazine showed the strongest synergistic effects, being even stronger as reported for LmqsRe28-1. Phage LmqsRe27-1 also showed synergistic effects with the three antibiotics tested, but these effects were less pronounced compared to the phage LmqsRe28-2. For the *P. aeruginosa* phage LmqsRe25-1, the strongest synergistic effects were observed in combination with trimethoprim-sulfadiazine as compared to combinations with enrofloxacin and gentamicin.

### Bacterial reduction and biofilm degradation capacity of phages

**Fig. 6:**
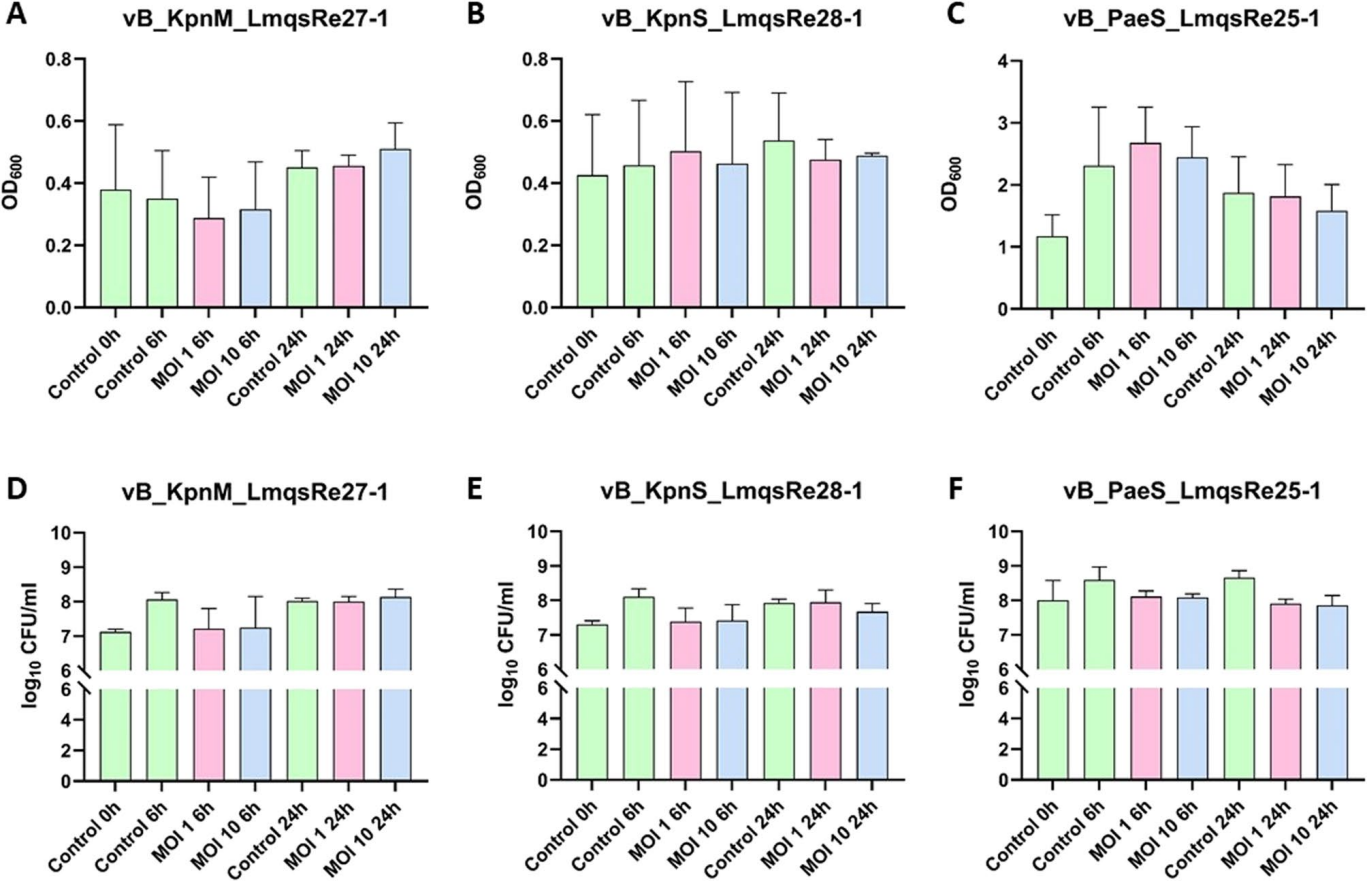
Optical density (OD_600_) of crystal violet stained biofilms (**A**-**C**) and bacterial concentration (**D**-**F**) as determined by counting of colony forming units (CFU) after plating biofilms produced by *Klebsiella pneumoniae* (A & D) isolate 12; B & E) isolate 2 and *Pseudomonas aeruginosa* (C & F) isolate 29) before incubation (Control 0 h; preformed biofilm (48 h old)), after 6 and 24 h with specific phages (multiplicity of infection (MOI) 1 and 10) and without specific phages (Control). No significant differences between groups and time were determined by Dunn’s multiple comparison test

After 48 h of incubation in microplate wells, biofilm production was observed for *P. aeruginosa* isolate 29 and *K. pneumoniae* isolates 2 and 12 but not for isolate 3. Therefore, LmqsRe28-2 – specific to *K. pneumonia* isolate 3 – was excluded from this experiment. The biofilm dispersion was determined by measuring optical density of crystal violet stain. Additionally, the number of viable bacteria was evaluated after co-incubation with phages by determining the CFU compared to an untreated control. Colony counts of *K. pneumoniae* isolate 2 showed 7.3 ± 0.1 log_10_ CFU/mL, *K. pneumonia* isolate 12 7.1 ± 0.1 log_10_ CFU/mL, and *P. aeruginosa* isolate 29 8.0 ± 0.6 log_10_ CFU/mL after 48 h of incubation. Similarly, co-incubation of preformed biofilms with phages at different MOIs did not result in a decrease in biofilm mass after 6 and 24 h (Fig. 6A-C). In addition, no significant effect of co-incubation with specific phages at different MOIs on bacterial growth (CFU) was observed at the same sampling points (6 and 24 h; Fig. 6D-F).

## Discussion

The present study aims to investigate possible contributions of phages to deal with to two major shortcomings of traditional antibiotic therapy in equine reproductive medicine, namely biofilm formation and antibiotic drug resistance in ESKAPE pathogens. After characterizing the newly isolated phages, their antibacterial activity was evaluated in vitro, in a biofilm model and in combination with selected antibiotic drugs.

Host ranges of the isolated phages were determined using clinical isolates of both bacterial species, all isolated from clinical horse samples. *Pseudomonas* phage LmqsRe25-1 showed plaque formation on 42% of the tested isolates (*n* = 33). *P. aeruginosa*-specific phages have been isolated and characterized in past studies [46–48], providing context for their host specificity. Two of the newly isolated *K. pneumoniae* phages (LmqsRe28-1 and LmqsRe28-2) were able to form plaques on 30 to 40% of tested isolates (*n* = 26). This is similar to previously characterized *Klebsiella* phages [44, 49–52], which lysed 22% [52] to 71% [51] of the tested bacterial isolates. Although combining the newly isolated phages in a phage cocktail [51] could have resulted in a broader lytic spectrum, experiments on cocktail design have not been intended to be part of this study but should be performed in future studies.

For further characterization, the phages with the broadest host range were selected and submitted to electron microscopy and whole genome sequencing. According to their morphological characteristics [53], phages were classified as podo- and siphoviruses [54]. Further genomic analysis confirmed the morphology as *Klebsiella* phages LmqsRe28-1 and LmqsRe28-2 were closely related to the unclassified *Caudoviricetes* phages *Klebsiella* phage P61_2 and *Klebsiella* phage vB_Kpn2-P1. For the third *K. pneumoniae*-specific phage, LmqsRe27-1, morphological characteristics (short tail, isometric head) were typical for podoviruses, confirmed by bioinformatics analysis showing the highest homologies with KP32i196 *Przondovirus* and presented with T7-like podovirus morphology (vB_KpnP_FZ12 [51]; K5-2 [55]; kpssk3 [56]). The *P. aeruginosa*-specific LmqsRe25-1 carries characteristics of siphoviruses in electron micrographs (non-contractile long tail, isometric head) [53]. The genome of LmqsRe25-1 revealed 98% identity with an unclassified *Pseudomonas* phage Meadow. Interestingly, in a recently published publication this *Pseudomonas*-specific transposable temperate phage showed lysogeny frequences of 26% in *Pseudomonas* isolate PA14 while 100% lysogeny frequence in isolate C0400 among survivors of phage challenge tests [44]. The low burst size and virulence index of LmqsRe25-1 are in coherence with the finding that this phage´s genome comprises lysogeny genes, excluding it from clinical use. Interestingly, the phages did not cluster in a host-species related manner according to phylogeny tree analysis based on the genome sequence of the phages and the phenotypically most similar phages LmqsRe28-1 and LmqsRe28-2 did not cluster when compared to the other examined phages.

Regarding the burst sizes and latent periods, large burst sizes and short latent periods are generally considered as indicators for highly efficient phages [57]. For the newly isolated phages, latent periods were comparable to *Klebsiella* and *Pseudomonas* phages isolated by others but burst sizes were significantly lower [51, 52, 58]. As the lysogenic character of LmqsRe25-1 is a possible explanation for the low burst size (< 1), absorption of phages to already phage-infected bacteria may lead to underestimation of burst sizes [59] – which might have occurred here since other parameters for phage fitness (e.g., host range, latent periods and virulence index) are within reported ranges [51, 52, 58].

Synergistic interaction of phages and antibiotics were tested in planktonic killing assays to identify highest efficacy panels. Gentamicin, enrofloxacin and trimethoprim-sulfadiazine were chosen as these are the most frequently used antibiotics to treat genital infections with *K. pneumoniae* and *P. aeruginosa* in horses [6]. In contrast to our observed benefit of gentamicin, a study by Vashisth et al. [60] concluded that gentamicin may reduce the lytic activity of bacteriophages by binding to the 16 s RNA at the 30 s ribosomal subunit, disrupting the bacterial mRNA translation [61]. The contrasting effect for gentamicin supplementation is likely due to the different bacterial species (*Acinetobacter baumannii*, *Staphylococcus aureus* and *Salmonella* Typhimurium) and their specific phages tested. The second antibiotic tested in this study, trimethoprim-sulfadiazine, is also an inhibitor of dihydrofolate reductase which is crucial for bacterial DNA-, RNA- and protein synthesis [62]. While one report observed an antagonistic effect of trimethoprim and phages against *E. coli* [45], the present study finds synergistic effects of trimethoprim-sulfadiazine and the newly characterized phages reported in the present study. This discrepancy is likely due to host-specific effects, as other groups have shown synergistic effects of trimethoprim-sulfadiazine-phage combinations against *K. pneumoniae* infections in other studies [63]. Enrofloxacin, the third antibiotic included, belongs to the class of gyrase inhibitors, and interferes with DNA replication [64]. In the present study, we observed synergistic effects of enrofloxacin-phage combinations against *K. pneumoniae* and *P. aeruginosa*, which is in line with other reports investigating the efficacy of fluorchinolone-phage (ciprofloxacin) combinations for inhibition of bacterial growth in vitro [44, 65]. Furthermore, synergistic effects have been observed in vivo for the combination of two phages and enrofloxacin for treating colibacillosis in broilers [66]. Though more research on the underlying mechanisms as well as the clinical robustness is needed to exploit phage-antibiotic synergies, a recently published case series holds promise for future clinical use of this therapeutic approach [67]. Our results provide the basic data on phages for further experiments in in vivo horse models.

In our study we tested the lytic activity of the newly isolated *Klebsiella* and *Pseudomonas* phages in a biofilm model. Biofilm-associated uterine infections are a major problem in equine endometritis [5] and several novel therapeutic approaches have been published in the last decade [67–69], adressing this issue. As biofilm degradation experiments in live animals have proven challenging [70], establishment of in vitro biofilm models [47, 71] and efforts to standardize these experiments are being underway [41]. Using the phages in our biofilm model, neither changes in biofilm density nor in viable bacteria were observed after co-incubation with specific phages against both bacterial species. Other studies have already demonstrated the benefit of phages for degradation of mature biofilms [47, 50, 72], but these results have not been confirmed by the novel isolated phages in the present study. This finding might be attributed to the lack of depolymerase genes, as determined by genomic analysis. Production of EPS-degrading enzymes is crucial for bacterial biofilms penetration [73] as they enable the phages to access deeper layers of biofilms [74]. For future experiments, depolymerase gene-carrying strains should be considered for treatment with a phage cocktail.

In conclusion, the present study reports the isolation and characterization of phages specific to the equine genital pathogens *K. pneumoniae* and *P. aeruginosa*. While the newly isolated phages were efficient in liquid culture, none of the tested phages was effective in the in vitro biofilm model. This might be attributed to the lack of depolymerases in our phages. Phage-antibiotic synergy for reducing bacterial growth in a liquid culture was observed even at antibiotic concentrations below the MIC. Thus, this multimodal approach shows suitability for the treatment of MDR infections in the future. However, the results of the present study are only a first step for future research, directed towards the in vivo therapeutic use of phages as an alternative or addition to traditional antibiotic therapy of genital infections in horses.

## Supplementary Information

Supplementary Material 1.

Supplementary Material 2.

Supplementary Material 3.

## Data Availability

The raw data supporting the conclusions of this article will be made available by the authors, without undue reservation due to reasons of sensitivity. Data are located in controlled access data storage at University of Veterinary Medicine Hannover, Foundation. The complete genomes of the isolated phages can be found under the accession numbers PV660644 (vB_KpnM_LmqsRe27-1), PV660645 (vB_KpnS_LmqsRe28-1), PV759140 (vB_KpnS_LmqsRe28-2) and (PaeS_LmqsRe25-1: partial genome) in the NCBI database (https://www.ncbi.nlm.nih.gov/).
