# Supplementary material for: Newly isolated bacteriophages show efficacy and phage-antibiotic synergy in vitro against the equine genital pathogens Klebsiella pneumoniae and Pseudomonas aeruginosa
Source: BMC Vet Res. 2025 Oct 3;21:568. doi: 10.1186/s12917-025-04989-1 (PMC12492686; doi:10.1186/s12917-025-04989-1)
Supplement: Supplementary file 2 — Supplementary Material 2. [file 12917_2025_4989_MOESM2_ESM.docx]

Supplementary Material: Fig. 1

1. **vB_Kpn_LmqsRe28-2
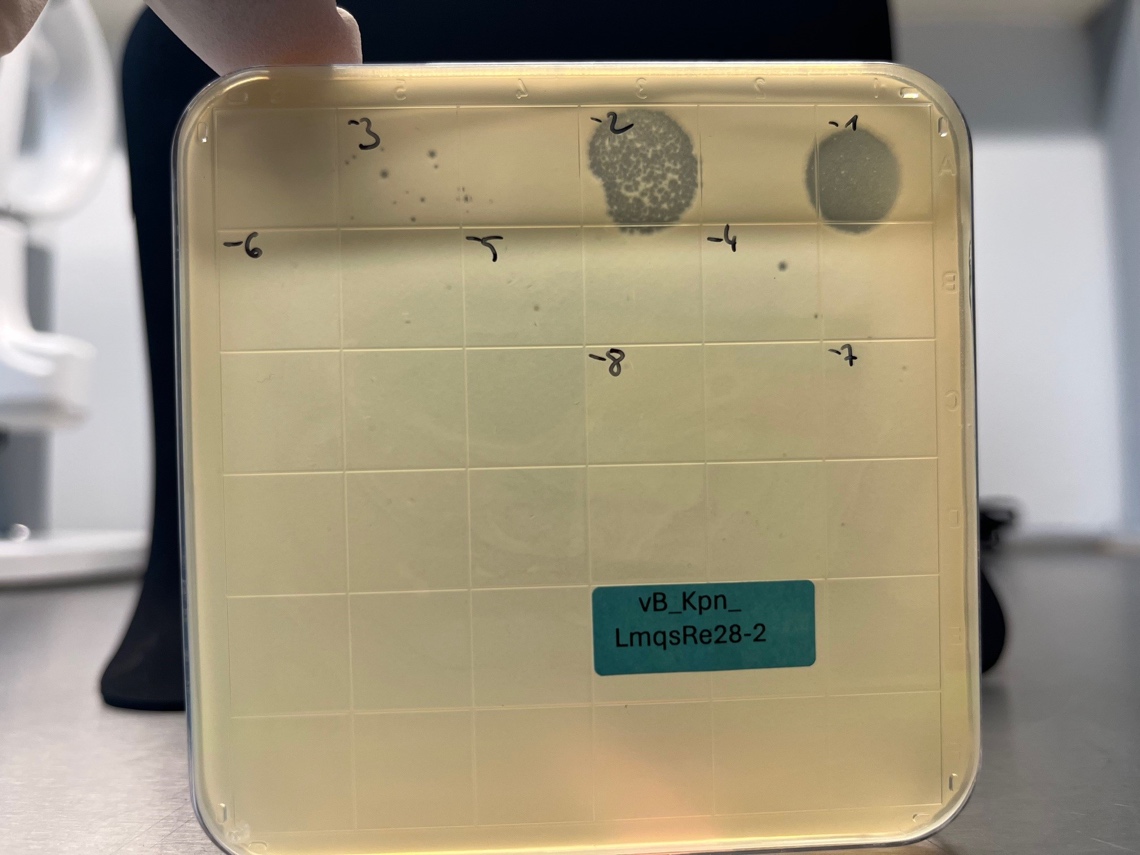
**
2. **vB_Kpn_LmqsRe28-1**

**
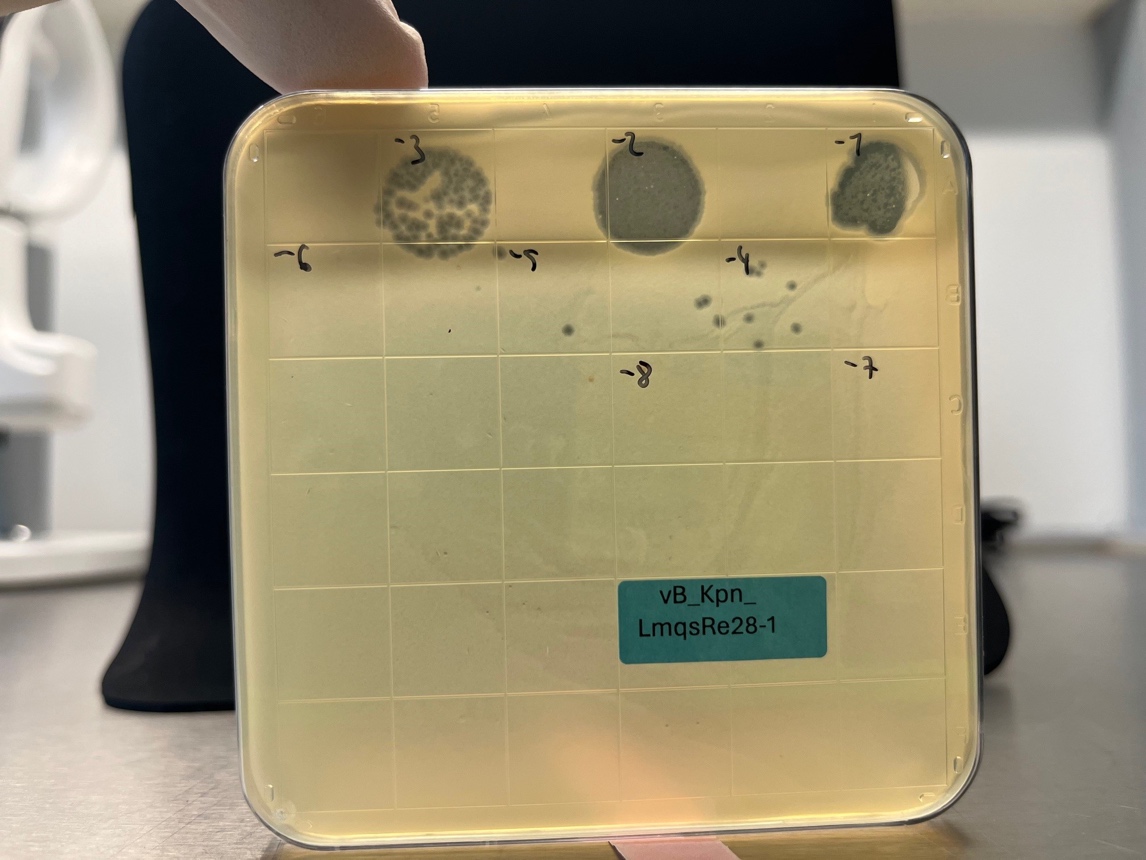
**

1. **vB_Kpn_LmqsRe27-1**
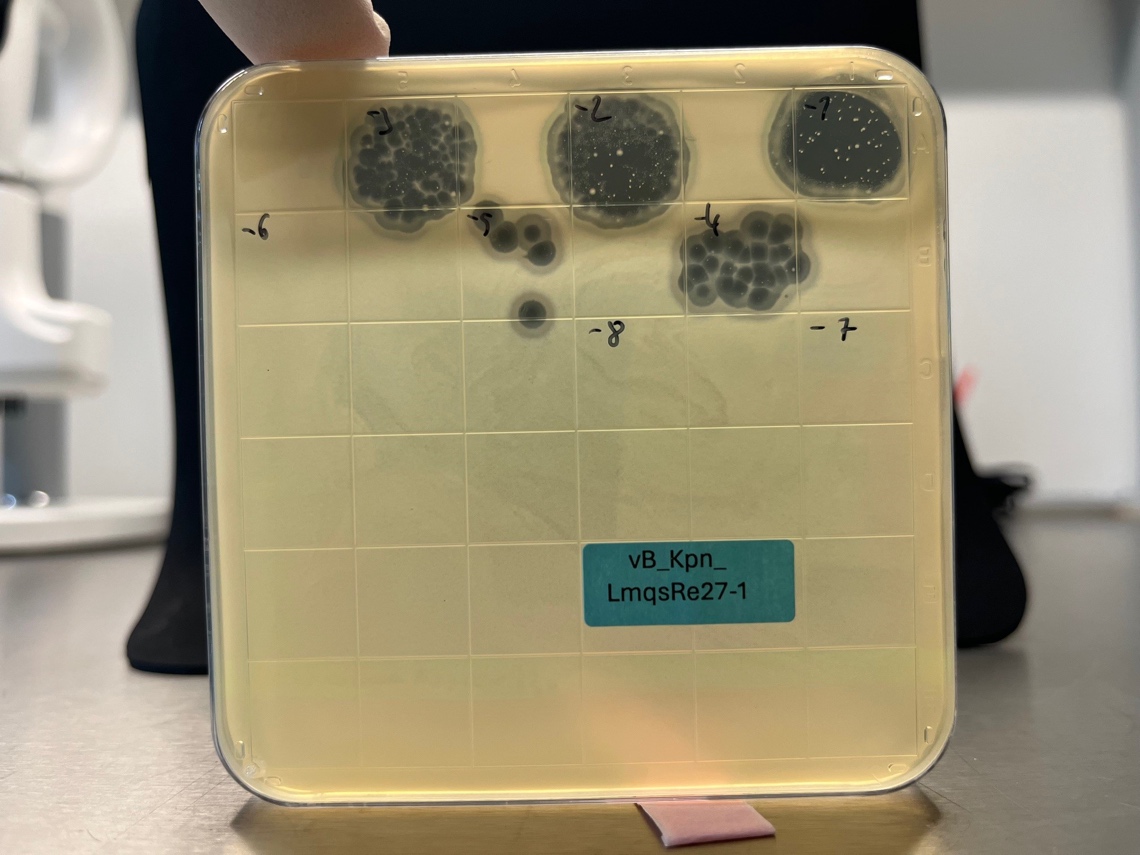

2. **vB_PaeS_LmqsRe25-1**


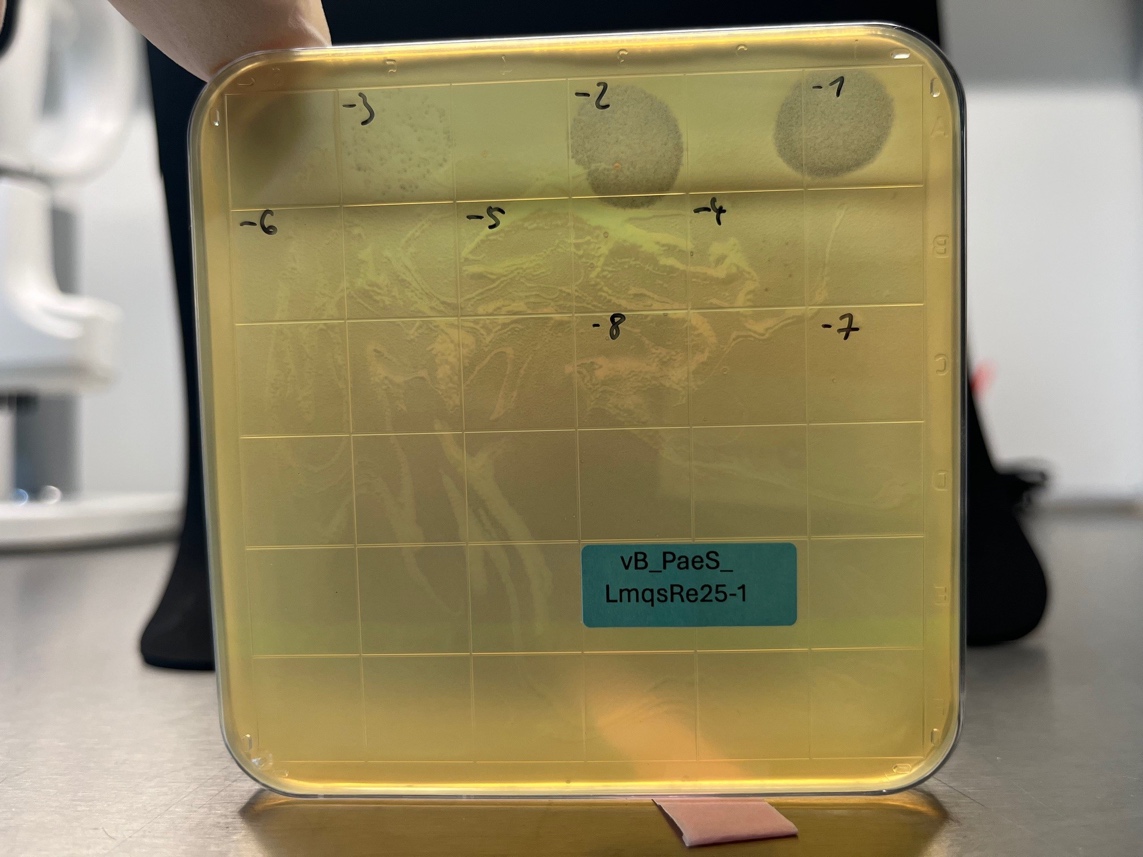


Supplementary Material Fig. 1: Plaque morphology of serially diluted phages vB_Kpn_LmqsRe28-2 (A), vB_Kpn_LmqsRe28-1 (B), vB_Kpn_LmqsRe27-1 (C) and vB_PaeS_LmqsRe25-1 (D) spotted on overlay plates with hosts *Klebsiella pneumoniae* Isolate 3 (A), *Klebsiella pneumoniae* Isolate 2 (B), *Klebsiella pneumoniae* Isolate 12 (C) and *Pseudomonas aeruginosa* Isolate 29 (D). Phages (100 µL of a 8-fold serial dilution series of the phage suspension) were plated on the respective host bacterial isolate using the soft agar-overlay technique. Images were taken after co-incubation for 24 h at 37°C.

Supplementary Material: Fig. 2

1. **vB_Kpn_LmqsRe28-2**

Burst size: 0.13465 ± 0.107961 PFU/cell Latent period: 30 min

1. **vB_Kpn_LmqsRe28-1**

Burst size: 1.337156 ± 2.003275591 PFU/cell Latent period: 30 min

1. **vB_Kpn_LmqsRe27-1**

Burst size: 47.69283 ± 41.96976 PFU/cell Latent period: 20 min

1. **vB_PaeS_LmqsRe25-1**

Burst size: 0.03 ± 0.010232 PFU/cell Latent period: 40 min

Supplementary Material Fig. 2: One-step Growth Curve of vB_Kpn_LmqsRe28-2 (A), vB_Kpn_LmqsRe28-1 (B), vB_Kpn_LmqsRe27-1 (C) and vB_PaeS_LmqsRe25-1 (D). One-step growth experiments at a multiplicity of infection of 0.02 using *Klebsiella pneumoniae* Isolate 3 (A), *Klebsiella pneumoniae* Isolate 2 (B), *Klebsiella pneumoniae* Isolate 12 (C) and *Pseudomonas aeruginosa* Isolate 29 (D) as bacterial hosts were performed. Each experiment was performed in triplicate and the average burst size (plaque forming units (PFU)/ cell) and latent period are given below growth curves.

Supplementary Material: Fig. 3


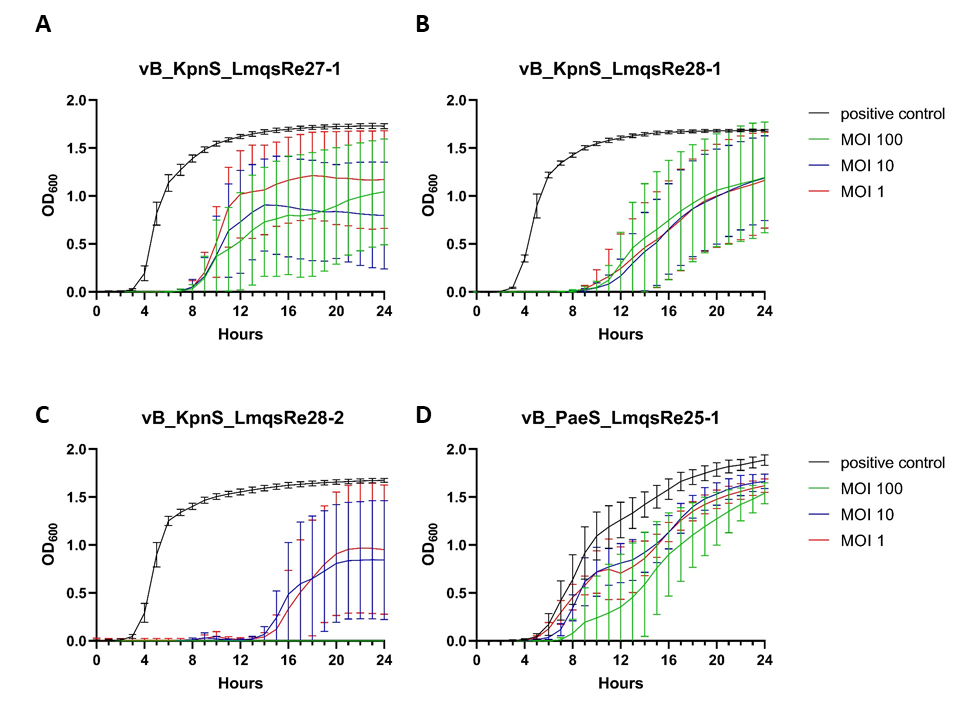


Supplementary Material Fig. 1. Growth curves of *Klebsiella pneumoniae* isolates (n=2; A: isolate 12, B-C: isolate 2) and *Pseudomonas aeruginosa* isolate 29 (n=1; D) incubated with different phages for 24h (A) vB_KpnM_LmqsRe27-1, B) vB_KpnS_LmqsRe28-1, C) vB_KpnS_LmqsRe28-2 and D) vB_PaeS_LmqsRe25-1 at different multiplicities of infection (MOI).
