# Supplementary material for: Newly isolated bacteriophages show efficacy and phage-antibiotic synergy in vitro against the equine genital pathogens Klebsiella pneumoniae and Pseudomonas aeruginosa
Source: BMC Vet Res. 2025 Oct 3;21:568. doi: 10.1186/s12917-025-04989-1 (PMC12492686; doi:10.1186/s12917-025-04989-1)
Supplement: Supplementary file 3 — Supplementary Material 3. [file 12917_2025_4989_MOESM3_ESM.docx]

Supplementary Material: Table 1

| Bacterial  isolate | Bacterial species | Origin of bacterial sample |
| --- | --- | --- |
| 1 | *K. pneumoniae* | endometrium, mare |
| 2 | *K. pneumoniae* | cervix, mare |
| 3 | *K. pneumoniae* | cervix, mare |
| 6 | *P. aeruginosa* | Sinus maxillaris rostralis, gelding |
| 7 | *P. aeruginosa* | tracheobronchial secretion, horse |
| 8 | *P. aeruginosa* | abdominal wound, horse |
| 12 | *K. pneumoniae* | wound secretion, horse |
| 15 | *K. pneumoniae* | urine, horse |
| 16 | *K. pneumoniae* | lung, horse |
| 27 | *K. pneumoniae* | cervix, mare |
| 28 | *K. pneumoniae* | cervix, mare |
| 29 | *P. aeruginosa* | wound, horse |
| 30 | *P. aeruginosa* | Fossa glandis, stallion |
| 31 | *P. aeruginosa* | Fossa glandis, stallion |
| 32 | *P. aeruginosa* | Fossa glandis, stallion |
| 39 | *K. pneumoniae* | umbilical abcessation, horse |
| 40 | *P. aeruginosa* | tracheobronchial secretion (horse) |
| 45 | *K. pneumoniae* | uterus, mare |
| 46 | *K. pneumoniae* | uterus, mare |
| 47 | *K. pneumoniae* | uterus, mare |
| 48 | *K. pneumoniae* | uterus, mare |
| 49 | *K. pneumoniae* | cervix, mare |
| 50 | *K. pneumoniae (ESBL)* | cervix, mare |
| 51 | *K. pneumoniae* | cervix, mare |
| 52 | *K. pneumoniae* | endometrium, mare |
| 53 | *K. pneumoniae* | endometrium, mare |
| 54 | *K. pneumoniae* | endometrium, mare |
| 55 | *K. pneumoniae* | endometrium, mare |
| 56 | *K. pneumoniae* | genital swab, mare |
| 57 | *K. pneumoniae* | genital swab, mare |
| 58 | *K. pneumoniae* | genital swab, mare |
| 59 | *K. pneumoniae* | genital swab, mare |
| 60 | *P. aeruginosa* | cervix, mare |
| 61 | *P. aeruginosa* | cervix, mare |
| 62 | *P. aeruginosa* | genital swab, mare |
| 63 | *P. aeruginosa* | Fossa glandis, stallion |
| 64 | *P. aeruginosa* | Fossa glandis, stallion |
| 65 | *P. aeruginosa* | genital swab, stallion |
| 66 | *P. aeruginosa* | Fossa clitoridis, mare |
| 67 | *P. aeruginosa* | respirational tract, horse |
| 68 | *P. aeruginosa* | wound secretion, horse |
| 69 | *P. aeruginosa* | bronchus, horse |
| 70 | *P. aeruginosa* | tracheobronchial secretion, horse |
| 71 | *P. aeruginosa* | ear, horse |
| 72 | *P. aeruginosa* | Apertura nasomaxillaris, horse |
| 73 | *P. aeruginosa* | hoof, horse |
| 84 | *P. aeruginosa* | cervix, mare |
| 85 | *P. aeruginosa* | cervix, mare |
| 86 | *P. aeruginosa* | genital swab, mare |
| 87 | *P. aeruginosa* | genital swab, mare |
| 88 | *P. aeruginosa* | Fossa clitoridis, mare |
| 89 | *P. aeruginosa* | tracheobronchial secretion (horse) |
| 90 | *P. aeruginosa* | tracheobronchial secretion (horse) |
| 91 | *P. aeruginosa* | nose left, horse |
| 92 | *P. aeruginosa* | guttural pouch, horse |
| 93 | *P. aeruginosa* | guttural pouch, horse |
| 94 | *P. aeruginosa* | larynx, horse |
| 95 | *P. aeruginosa* | infected abdominal suture, horse |
| 96 | *K. pneumoniae* | cervix, mare |
| 97 | *K. pneumoniae* | cervix, mare |
